# The immune response of T cells and therapeutic targets related to regulating the levels of T helper cells after ischaemic stroke

**DOI:** 10.1186/s12974-020-02057-z

**Published:** 2021-01-18

**Authors:** Tian-Yu Lei, Ying-Ze Ye, Xi-Qun Zhu, Daniel Smerin, Li-Juan Gu, Xiao-Xing Xiong, Hong-Fei Zhang, Zhi-Hong Jian

**Affiliations:** 1 Department of Neurosurgery, Renmin Hospital of Wuhan University, Wuhan, 430060 Hubei Province People’s Republic of China; 2 Central Laboratory, Renmin Hospital of Wuhan University, Wuhan, 430060 Hubei Province People’s Republic of China; 3 Department of Head and Neck and Neurosurgery, Hubei Cancer Hospital, Wuhan, 430079 Hubei Province People’s Republic of China; 4 University of Central Florida College of Medicine, Orlando, FL 32827 USA; 5 Department of Anesthesiology, Zhujiang Hospital of Southern Medical University, Guangzhou, Guangdong People’s Republic of China

**Keywords:** T cell subsets, Immune responses, Ischaemic stroke

## Abstract

Through considerable effort in research and clinical studies, the immune system has been identified as a participant in the onset and progression of brain injury after ischaemic stroke. Due to the involvement of all types of immune cells, the roles of the immune system in stroke pathology and associated effects are complicated. Past research concentrated on the functions of monocytes and neutrophils in the pathogenesis of ischaemic stroke and tried to demonstrate the mechanisms of tissue injury and protection involving these immune cells. Within the past several years, an increasing number of studies have elucidated the vital functions of T cells in the innate and adaptive immune responses in both the acute and chronic phases of ischaemic stroke. Recently, the phenotypes of T cells with proinflammatory or anti-inflammatory function have been demonstrated in detail. T cells with distinctive phenotypes can also influence cerebral inflammation through various pathways, such as regulating the immune response, interacting with brain-resident immune cells and modulating neurogenesis and angiogenesis during different phases following stroke. In view of the limited treatment options available following stroke other than tissue plasminogen activator therapy, understanding the function of immune responses, especially T cell responses, in the post-stroke recovery period can provide a new therapeutic direction. Here, we discuss the different functions and temporal evolution of T cells with different phenotypes during the acute and chronic phases of ischaemic stroke. We suggest that modulating the balance between the proinflammatory and anti-inflammatory functions of T cells with distinct phenotypes may become a potential therapeutic approach that reduces the mortality and improves the functional outcomes and prognosis of patients suffering from ischaemic stroke.

## Introduction

Stroke is not only one of the main causes of death but also the primary cause of long-term disability worldwide; however, extensive therapeutic options are lacking, which creates a dominating economic and medical burden [1]. Ischaemic stroke results from the blockade of the blood vessels supplying the brain, accounting for 87% of all strokes in the USA [1] and is currently the main focus of stroke research.

Stroke can occur at any age but mostly occurs at an older age (beyond 65 years old) [2]. Elderly patients have an elevated risk of complications and worse outcomes after treatment compared with younger patients, partially due to alterations in the immunological response to stroke [3]. Women are more vulnerable to stroke after menopause than before menopause due to the lack of female gonadal hormone protection, which may regulate T cells [4].

Despite numerous factors affecting the onset and progression of brain injury after stroke, the consistent, basic process is intimately connected with the immune response, including T cell responses. In the brain of healthy people, only a few T cells enter the central nervous system (CNS) and are found in the parenchyma, perivascular space and cerebrospinal fluid (CSF) due to the intact blood-brain barrier (BBB). These cells perform immune surveillance to maintain CNS homeostasis in cooperation with CNS-resident immune cells [5]. After stroke onset, the acute cessation of the blood supply induces primary irreversible tissue injury and results in neural cell death, the site of which constitutes the ischaemia core; neural cell death results in a subsequent release of damage-associated molecular patterns (DAMPs). The ensuing brain injury that damages the peri-infarct area (the penumbra) is caused by a rapid cascade of events such as excitotoxicity, oxidative stress and mitochondrial disturbance [6]. In the process of neural cell death, different cellular signalling pathways that regulate autophagy and apoptotic cell death (Mst1, ULK1, Bax, Caspase-3 and Bcl-2), necroptotic cell death (TRAF2 and RIPK1/RIPK3/MLKL) [7], the cellular metabolic state (TSC1/TSC2, p-mTOR, and mTORC1), the oxidative defence system (FoxO1, β-catenin/Wnt, and Yap1) and inflammatory reactions (jak2/stat3 and Adamts-1) are changed [8–10]. However, the cellular signalling pathways related to jak2/stat3 and Adamts-1 involved in regulating inflammatory reactions are found to be predominantly localized in macrophages/microglia [9] in the post-ischaemic brain, which may account for the fact that these pathways first trigger inflammation in brain-resident immune cells, including microglia and macrophages [11], after ischaemic stroke onset. With the release of inflammatory factors, cytokines, chemokines and DAMPs, a large number of peripheral immune cells infiltrating the injured site participate in innate and adaptive immune responses. Additionally, neutrophils, monocytes and CD8^+^ cells are regarded as the first peripheral immune cells to invade the injured brain within hours after stroke onset [11]. Subsequently, CD4^+^ cells are reported to infiltrate the brain approximately 24 h after ischaemia [11]. Regulatory T (Treg) cells remain in the injured brain for more than 30 days after ischaemia to control the aggravation of inflammation by regulating the levels of inflammatory factors [12]. Nonetheless, the roles of T cells in tissue damage and repair have not been completely elucidated.

**Fig. 1:**
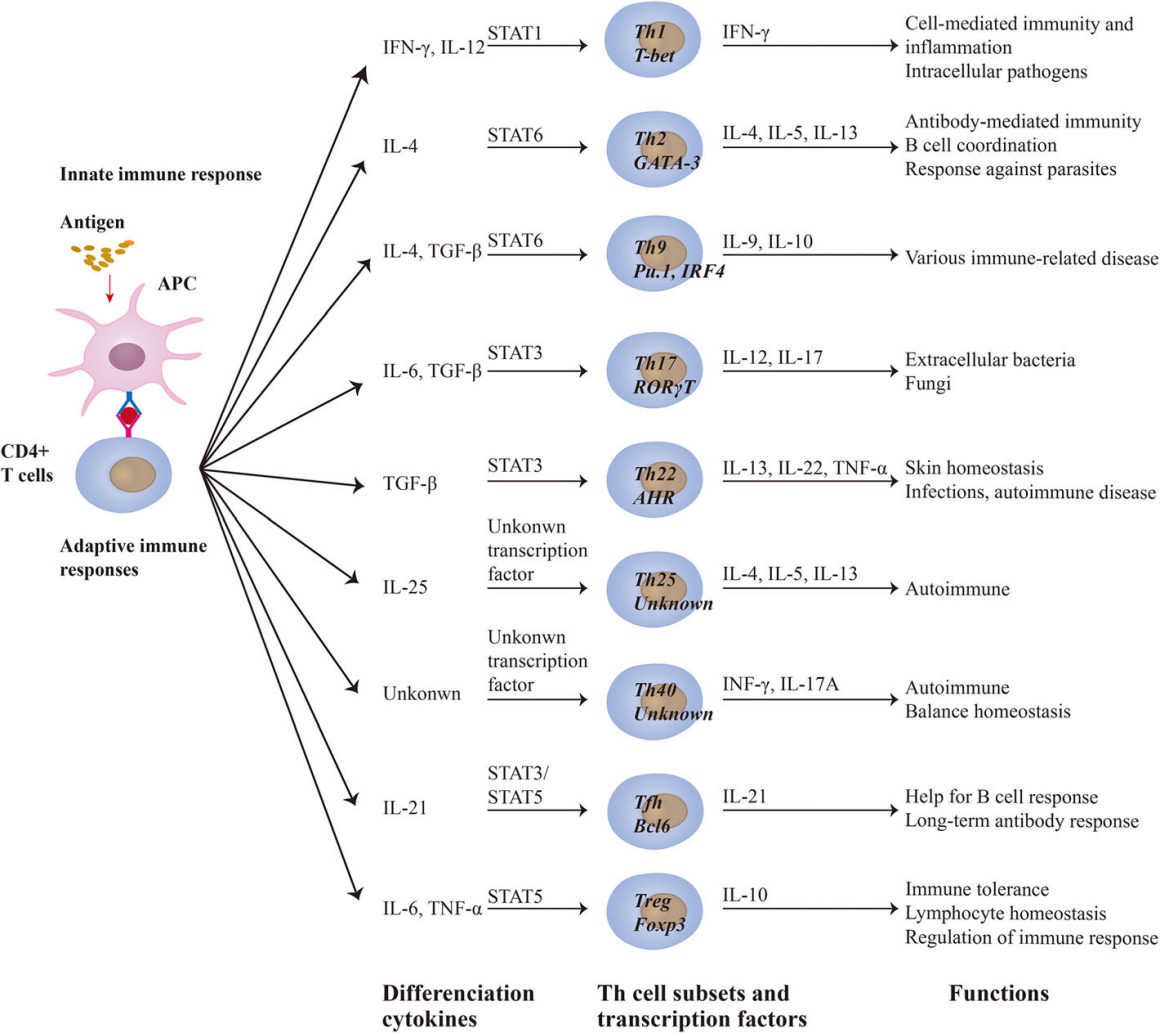
Functions of Th cell subsets

Over the past 20 years, a number of studies have considered the immune system to have vital effects on the process and development of brain injury following ischaemia, especially the recruitment and function of macrophages and neutrophils rather than T cells in the injured brain. However, recently, due to the variety of T cell types, an increasing number of researchers have found T cells to be vitally involved in the onset and progression of ischaemic stroke because these cells not only promote the occurrence of inflammation by infiltrating the injured brain in the early stages after ischaemic stroke [13] but also exert effects on repair and functional improvement in the late stage after ischaemic stroke [14]. T helper (Th) 1 and Th2 cells were the first subsets of Th cells identified [15]. Then, Th17 cells, which differentiate in the presence of both TGF-β and IL-6, were recognized to generate proinflammatory cytokines [16]. The expression of Foxp3 is a characteristic of Treg cells that exert immunomodulatory functions [17]. Follicular T helper (Tfh) cells in the follicles were found to facilitate B cell responses [18]. Some subsets of T cells, such as Th9, Th22 and Th25 cells, are recognized by the production of different cytokines (expressing IL-9, IL-22 and IL-25, respectively) [19–21]. CD4loCD40+ T cells, recognized as Th40 cells, were found to greatly expand under autoimmune conditions and play a vital role in type 1 diabetes [22]. The diverse subsets of T cells are simply presented in Fig. 1.

Here, we described the different subsets of T cells and identified several vital T cell subsets related to acute and chronic processes during ischaemic stroke, especially Th1, Th2, Th17, γδ T, Treg and even Th40 cells. We also reported several very promising therapeutic targets related to the modulation of T cell responses to improve the strengths of immune responses in the injured brain. Further research towards understanding the mechanisms modulating the emergence of cerebral inflammation mediated by T cell subsets may facilitate the development of a novel or adjunctive way to treat stroke.

## Subsets and plasticity of Th cells

CD4^+^ cells (Th cells) exert distinct effects during the immune response. Remarkably, Th cells have the capability of differentiating into subsets with functionally diverse phenotypes, such as Th1, Th2, Th9, Th17, Th22, Th25, Th40, Tfh and Treg cells, in response to different cytokine milieus and antigen stimulation [23, 24]. It is widely known that the differentiation of Th1 cells is induced by successive responses to interferon (IFN)-g and interleukin (IL)-12, which are initiated by coordinated signalling through the communication between STAT1-associated cytokine receptors and T cell receptor (TCR) [25]. Th1 cells have been shown to enhance and promote the elimination of certain intracellular pathogens [15]. Through the interaction between TCR signalling and IL-4 receptor signalling mediated by STAT6, which may react synergistically to upregulate low expression of GATA-3, a subset-determining transcription factor of Th2 cells, Th2 differentiation is induced [26], and Th2 cells function to enhance the clearance of parasites [15]. Th17 cells generate proinflammatory effector cytokines, including IL-17A, IL-17F, IL-22 and IL-26; of these cytokines, IL-17A and IL-17F modulate tissue inflammation by inducing the secretion of proinflammatory cytokines and chemokines, such as IL-1β and IL-6 [27, 28]. Compared with their proinflammatory role, the regulatory role of Th17 cells, which involves suppressing Th17-induced inflammation, may be linked with IL-10 and IL-21 by Foxp3^+^ Treg cells and type 1 regulatory T (Tr1) cells [29]. It has been reported that exposing effector cells to TGF-β and IL-6 sustainably drives the production of IL-17 and IL-10, which is important in modulating the level of the potentially detrimental Th17 cell-mediated immune response in an experimental autoimmune encephalomyelitis (EAE) model [30]. Th9 cells are a newly discovered CD4^+^ Th cell subset that mainly secrete the lineage-specific cytokine IL-9 [19, 31]. Based on the discovery study, Th9 cells have indispensable roles in the initiation and development of immune responses [32]. Generally, Th9 cells are proinflammatory and participate in various immune-related diseases, highlighting their pathological roles in inflammation and the immune system [33]. However, Th9 cells are also reported to have a protective effect on parasitic infections, indicating that these cells have various functions [33]. Th22 cells are localized in the skin by their high expression of CCR4, CCR6 and CCR10 [20, 34], and IL-22 has been shown to activate innate immune responses against pathogenic bacteria, especially in respiratory and gut epithelial cells [35], which indicates their essential roles in skin homeostasis and skin disease pathogenesis. Importantly, it has been reported that activation of AHR, a subset-determining transcription factor of Th22 cells, after acute ischaemic stroke plays a vital role in brain ischaemic injury by modulating astrogliosis and neurogenesis [36]. In addition, Th22 cells are closely related to multiple types of diseases, such as infections, autoimmune diseases, hepatitis, pancreatitis, rheumatoid arthritis (RA) and tumours, because of the wide distribution of IL-22R [37]. The Th25 cell-secreted cytokine IL-25 was reported to be related to Th2 responses [21]. Furthermore, IL-4, IL-5 and IL-13 gene expression induced by IL-25 results in a Th2-like response characterized by increased serum levels of IgE, IgG1 and IgA; blood eosinophilia; and a series of pathological changes, including infiltration of eosinophils, increased production of mucus and hyperplasia/hypertrophy of epithelial cells in the lungs and digestive tract and participates in the clinical evolution of leprosy in cooperation with the Th2 cytokine profile [35, 38, 39].

When CD40 is uniquely expressed on T cells, distinct from the receptor expressed on antigen-presenting cells (APCs), it serves as a functional receptor on T cells, representing a new T cell subset [40]. Studies have found that Th40 cells (CD4loCD40+ T cells) produce IFN-γ (signal of Th1 cells) and IL-17A (signal of Th17 cells) [41], are proinflammatory and balance Treg cells to maintain a homeostatic state and that Th40 cells from healthy bodies are able to produce regulatory Th2 cytokines to control autoimmunity, while pathogenic Th40 cells expand quickly throughout the progression of diabetes [42, 43]. Th40 cells not only have vital effects in autoimmunity [44] but also importantly play a pivotal role in the injured brain after cardiac arrest and cardiopulmonary resuscitation (CA/CPR) in mouse models [45]. Tfh cells, which are characterized by their expression of CXCR5 [18, 46], a B cell homing chemokine CXCL13 receptor, are a novel subset of T cells that play essential roles in facilitating B cell responses, including B cell affinity maturation, class switch recombination and plasma and memory B cell maintenance for humoral memory [47–49]. Studies have also verified that both Tfh cells and CD8^+^ T cells share key features of a memory cell precursor gene expression programme containing Bcl6 and IL-17Rα, indicating that Tfh cells have the capacity to form memory early [50]. However, they have not been researched in stroke.

Treg cells are divided into two populations, Foxp3^+^ Treg cells and Foxp3^−^ Treg cells, the latter of which includes Th3 and Tr1 cells [51]. Foxp3^+^ Treg cells exert vital functions involved in downregulating the pathological T cell response through secretion of the anti-inflammatory cytokines IL-10 and TGF-β [17, 52], while Tr1 cells play their regulatory role by affecting different target cells, including effector CD4^+^ and CD8^+^ T cells, myeloid APCs and B cells [53]. There are also interactions between the two types of Treg cells, and it has been demonstrated that Foxp3^+^ Treg cells are required for the initial stage of inflammatory target organ tolerance induction, while Tr1 cells have an important effect on the maintenance of long-lasting tolerance [54, 55]. Recently, numerous studies have revealed the mechanisms underlying Treg cell participation in tissue regeneration, which occurs not only through tissue-specific anti-inflammatory effects but also through direct regenerative mechanisms [56, 57]. Th3 cells primarily secrete TGF-β, which is distinct from TGF-β secreted by Th2 cells, to provide help for IgA and suppress functions of Th1 and other immune cells in the gut [58]. Th3 cells are mainly induced by oral antigens, and enhanced differentiation from Th0 precursors can be achieved by culture with TGF-β, IL-4, IL-10 and anti-IL-12 antibodies [59]. Furthermore, Th3 cells secreting TGF-β are capable of suppressing systemic autoimmune and inflammatory responses to treat related diseases [60]. Tr1 cells generate immunosuppressive cytokines, including IL-10 and TGF-β, to inhibit the effects of effector immune cells and are reported to be induced upon antigen exposure [53]. Additional studies have tested the protective role of Tr1 cells in various mouse models, including models of multiple sclerosis, intestinal inflammation, EAE and even NOD mice with diabetes [61]. Nasal tolerance in models of atherosclerosis, cardiac ischaemia, lupus and stroke has also been identified [62]. Mucosal antigens have also been found to effectively treat animal models of stroke, but the underlying mechanisms remain to be elaborated [63]. In conclusion, recent reports indicate that endogenous Treg cells exert a neuroprotective function by secreting TGF-β and IL-10 and have a protective effect against ischaemic brain injury by increasing the number of Treg cells in the circulation [64]. Therefore, the function of Treg cells after ischaemia needs to be explored in depth.

Although there are many diverse subsets of CD4^+^ T cells, the plasticity of Th cells has been extensively reported and is contrary to previous views stating that the functions of distinct T cell subsets are irreversible. Initially, Th1, Th17, Th22 and Th40 cells were classified as a group that predominantly protects against extracellular pathogens by secreting cytokines with the same function, while Th2, Th9 and Th25 cells were thought to mainly exert functions in autoimmune disease and allergic inflammation [39, 65, 66]. Notably, it appears that induced Treg (iTreg) cells (induced in the periphery) and Th17 cells may be relatively unstable and have flexibility in their differentiation and function because of the unstable expression of Foxp3 by iTreg cells and that of IL-17 by Th17 cells [67, 68]. Th17 cells have been shown to transdifferentiate into Treg cells by altering their characteristic transcriptional profile and acquiring potent regulatory properties through the effects of TGF-β signalling and AHR, indicating that Th17 cells may secrete anti-inflammatory cytokines to attenuate inflammation [69]. Likewise, Th17 cells have been reported to shift into nonclassical Th1 cells and Th2 cells in the presence of IL-12 or IL-4, respectively [70, 71]. Treg cells have been demonstrated to be capable of becoming Th1 cells in the presence of IL-12 in vitro and attaining Treg-Th17 plasticity in the presence of IL-6 and TGF-β [72, 73]. There are other transitions, such as Th2 cells transforming into Th9 cells and Th0 cells in response to TGF-β or IL-12/IFNs, respectively, and Th9 cells converting into the Th1 phenotype and subsequently producing IFN-γ in vivo [74–76]. That means that some cell subsets can transform each other to maintain homeostasis. These findings provide novel insight into the immune response of T cells after ischaemic stroke and a more comprehensive understanding of the functions of T cell subsets.

## T cells and ischaemic stroke (Fig. 2)

### Spatial and temporal features of T cell responses after ischaemia

Several studies using recombination activation gene (Rag)-deficient mice and severe combined immunodeficiency (SCID) mice, which both lack T cells and B cells, have shown significantly reduced infarct volumes and lower neurological deficits in these mice compared with the corresponding wild-type mice, regardless of the stroke induction model studied. By comparing *Rag1*^−/−^ mice reconstituted with B cells to *Rag1*^−/−^ mice reconstituted with CD3^+^ T cells, studies have indicated that T cells likely have a vital effect on early stroke evolution and exert a detrimental effect as early as 24 h after stroke through an antigen-independent mechanism [77–79]. Our study showed that the protective roles of T cell deficiency in brain injury after ischaemic stroke were relevant to transient middle cerebral artery occlusion (tMCAO) in a rodent model, indicating that reperfusion after ischaemic stroke might be closely related to T cell responses [80]. Additionally, a study found that depletion of CD4^+^ T cells or CD8^+^ T cells reduced infarct volume in late stages after tMCAO [81]. Different studies and results indicate that distinct T cell subsets invade the brain dynamically and play various roles in the different stages after experimental ischaemic stroke (Table 1). Studies have shown that T cell infiltration occurs from hours to 30 days after stroke. Researchers have not arrived at a consistent or definite conclusion on the time of peak T cell infiltration into the injured brain after stroke.

**Fig. 2:**
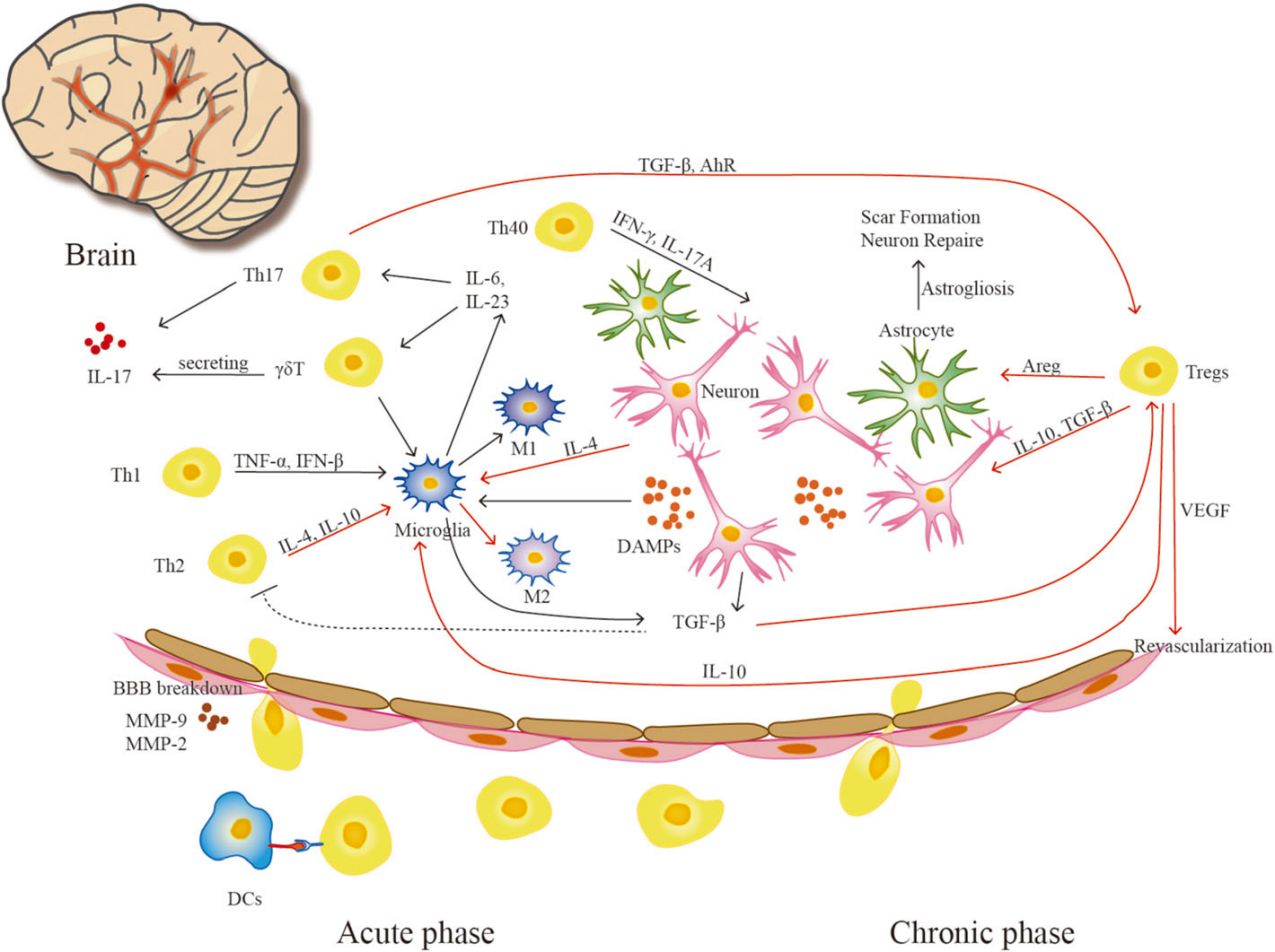
Functions of T cell subsets in the acute and chronic phases after ischaemic stroke. In the acute phase, necrotic cells release DAMPs to activate brain-resident microglia, and then activated microglia secrete cytokines, such as IL-23, to recruit γδ T cells via an antigen-independent pathway. IL-17 released by γδ T cells, other inflammatory cytokines and antigens from the injured brain induce antigen-dependent immune responses by Th1, Th2, Th17 and Th40 cells. Th2 cells exert a protective role by promoting microglial M2 polarization. Other cells interact with M1 microglia to play detrimental roles in the injured brain or secrete proinflammatory cytokines. In the chronic phase, Treg cells may exist until 1 month after stroke onset and perform roles in recovery via several pathways, including scar formation, neuronal repair and revascularization

**Table 1:** Temporal T cell responses in different models

Author	Animal model	T cell types	Peak time	Year
Yilmaz [82]	60 min MCAO	CD4^+^ T cell	24 h	2006
Hurn [83]	90 min MCAO	Spleen T cell	22 h	2007
Gelderblom [84]	60 min MCAO	CD4^+^ T cell	3 days	2009
	60 min MCAO	CD4^−^CD8^−^ lymphocytes	1/3 days	2009
Kleinschnitz [85]	60 min MCAO	CD4^+^ T cell	24 h	2010
Brait [86]	30 min MCAO	CD3^+^ T cell	1 days	2010
Saino [87]	pMCAO	CD4^+^ T cell	3 h	2010
Deng [57]	6/8 min CA/CPR	T cell	3 h	2014
Liesz [88]	pMCAO	CD3^+^ T cell	7/14 days	2013
Stubbe [7]	30 min MCAO	Treg	14/30 days	2013
Vindegaard [89]	pMCAO	CD3^+^ T cell	14/28 days	2017
Xie [90]	90 min MCAO	CD4^+^ and CD8^+^ T cell	1 month	2018

Gelderblom et al. found that T lymphocyte numbers were increased in the infarcted hemisphere on day 3 after experimental ischaemia reperfusion (I/R) and reported that significant infiltration of CD4^−^/CD8^−^ lymphocytes occurred on days 1 and 3, while the infiltration of CD4^+^ cells increased on day 3 after experimental I/R [91]. Additional studies demonstrated that T lymphocytes (CD3^+^ cells) significantly increased in number within the cerebral infarct and in peri-infarct areas, with significantly fewer T lymphocytes in the spleen at 24 h after experimental I/R, while the number of T lymphocytes in the circulation was unchanged at the same time [77, 92, 93]. Our studies also found that stroke could induce lymphopenia and reduce splenocyte T cell numbers due to the roles of T cells at 48 or 72 h after stroke [94, 95]. Saino et al. found that CD4^+^ cells infiltrated the infarct cortex within 3 h after stroke [96]. Another study demonstrated that Th40 lymphocyte numbers were elevated 3 h after cardiac arrest, a cause of ischaemic stroke, and CA/CPR, decreased greatly in 24 h, and increased again on day 2 and day 3 [45].

Liesz and co-workers used spectratype/immunoscope analysis to demonstrate that the number of CD3^+^ T cells peaked at 7 days after permanent middle cerebral artery occlusion (pMCAO) and that clonal T cell expansion occurred in T cells isolated from the ipsilateral brain at 7 days after pMCAO and in T cells isolated from the spleen at 14 days after pMCAO [82]. Moreover, Xie et al. showed that CD4^+^ and CD8^+^ T cells exhibited prolonged activation after experimental ischaemic stroke, indicating that these cells had a greater role in neuronal repair than Treg cells [85]. Vindegaard and Munoz-Briones found an obvious increase in CD3^+^ T cell numbers within the ipsilateral brain, not only within the infarct core but also within the corpus callosum on days 14 and 28 after pMCAO [97]. Stubbe et al. demonstrated that Treg cells accumulated and proliferated in the ipsilateral brain on days 14 and 30 post-stroke and were accompanied by increases in the number and activation of microglia after tMCAO [12]. In two previous studies, the authors demonstrated an obvious increase in T cell infiltration in the injured brain on day 7 through day 30 or 60 of the post-stroke inflammatory response in rodents after cerebrocortical photothrombosis or pMCAO, respectively [98, 99]. These results show that T cells may exert a detrimental function in the early stage while playing a protective role in the late stage of the inflammatory response after ischaemic stroke. However, the mechanisms and functions of T cells in the CNS remain to be clarified (Table 2). Although we know that Th cells exert different functions at different stages after stroke, Th cells are divided into several subsets, and we have to clarify which of these subsets play a dominant role during the onset and development of stroke.

**Table 2:** Characteristic of T cells infiltrating into the injured brain in acute and chronic phases after

Author	Animal model	Acute phase (within 7 days)	Chronic phase (after 14 days)	Year
Stubbe et al. [7]	30 min MCAO	CD4^+^ T cells and Tregs elevating in the peri-infarct and infarct area with MHCII+ DCs and MHCII+ macrophages after ischemic stroke	More CD4^+^ and Tregs elevating in the ischemic hemisphere consistent with increasing MHCII+ microglia, DCs and macrophages in the injured brain after ischemic stroke	2013
Vindegaard et al. [89]	pMCAO	Only a few CD3^+^ T cells infiltrating into the brain, predominantly located to the meningeal areas or in close proximity to a vessel, in the vicinity of the infarct with a few macrophage/microglia infiltrating the infarct area	A high number of macrophage/microglia infiltrating the infarct area and increasing T cell numbers within the infarct core and the corpus callosum	2017
Xie et al. [90]	90 min MCAO	Activated/memory phenotype of T cells (either CD4^+^ or CD8^+^) infiltrating the ischemic hemisphere	Greater proportion of activated/memory T cells than the acute phase with CD25, a T cell activation antigen, increasing in both brain-invading CD4^+^ and CD4^−^ T cells	2018

### Different T cell subsets infiltrating the injured brain (Fig. 3)

#### Th1 and Th2 cells related to brain injury

We identified that Th1 and Th2 cells have vital effects on the early phase of the post-stroke inflammatory response by evaluating the infarct sizes and neurological scores 2 days after stroke in a Th1- and Th2 cell-deficient mouse model [84]. Th1 cells and Th2 cells have different impacts on ischaemic brain injury. Th1 cells secrete proinflammatory cytokines, such as IFN-γ and chemokines, and produce numerous reactive oxygen species (ROS) and nitric oxide to destroy the BBB. Th2 cells secrete anti-inflammatory cytokines, such as IL-4, IL-10 and IL-13, to promote nerve growth factor (NGF) production, debris removal, tissue remodelling and repair, and angiogenesis after brain ischaemia [83, 86]. Interestingly, Th1/Th17 cells and Th2 cells separately perform crosstalk with M1 and M2 microglia [100]. Initially, microglia are derived from macrophages that undergo migration and differentiation in the process of original haematopoiesis in the foetal yolk sac, and then they are localized in the brain with the ability to proliferate in the process of neonatal growth, while granulocyte-monocyte progenitors are the precursors of macrophages in the periods of development and adulthood [87, 101]. Both microglia-derived macrophages (MiDM) and monocyte-derived macrophages (MoDM) in the injured brain show the capabilities of polarizing into a proinflammatory or anti-inflammatory (M1 or M2, respectively) phenotype and performing phagocytosis function, as well as exhibiting a high degree of morphological plasticity [101]. However, MiDM perform more vital roles due to their abilities to facilitate neuronal viability and modulate neuronal excitability as well as secrete NGF [88]. At the same time, activation of MiDM relies on ATP/ADP signalling, which may account for the number of MiDM being influenced by energy deficiency and alterations in local blood perfusion [89, 90]. Th1 cells can promote M1 polarization through the induction of proinflammatory cytokines, including TNF-α and IFN-γ [102]. M1 microglia also induce and recruit Th1 cells by secreting IL-12 and TNF-α and expressing chemokines, such as CXCL9 and CXCL10 [103]. Th2 cells promote M2 polarization by secreting anti-inflammatory cytokines (IL-4, IL-10 and IL-13) [102, 104, 105] and increase the levels of insulin-like growth factors, neurotrophic factors secreted by microglia, to augment the neuroprotective role of microglia [83, 106, 107]. M2 cells can induce and recruit Th2 cells by secreting IL-4, CCL17, CCL22 and CCL24 [102, 103]. Th17 cells have also been shown to cause brain injury through crosstalk with M1 microglia via secreted IL-17 [100]. A recent study found that Th2/Th17 cells could enhance blood perfusion in ischaemic injury by regulating angiogenesis and inducing endothelial sprouting [108]. However, there are studies implying that M1/M2 cells have complicated roles in view of the complexity and diversity of M2 subtypes [74] and the phenotypic transformation between M2 and M1; these studies have identified that both M1 cells and M2 cells have proinflammatory and anti-inflammatory functions [101] rather than oversimplified single functions and that the functions of these cells are more complex in vivo than in vitro and are harder to study in humans than in mice. Whether and how these functions occur after ischaemic stroke remain to be elucidated.

**Fig. 3:**
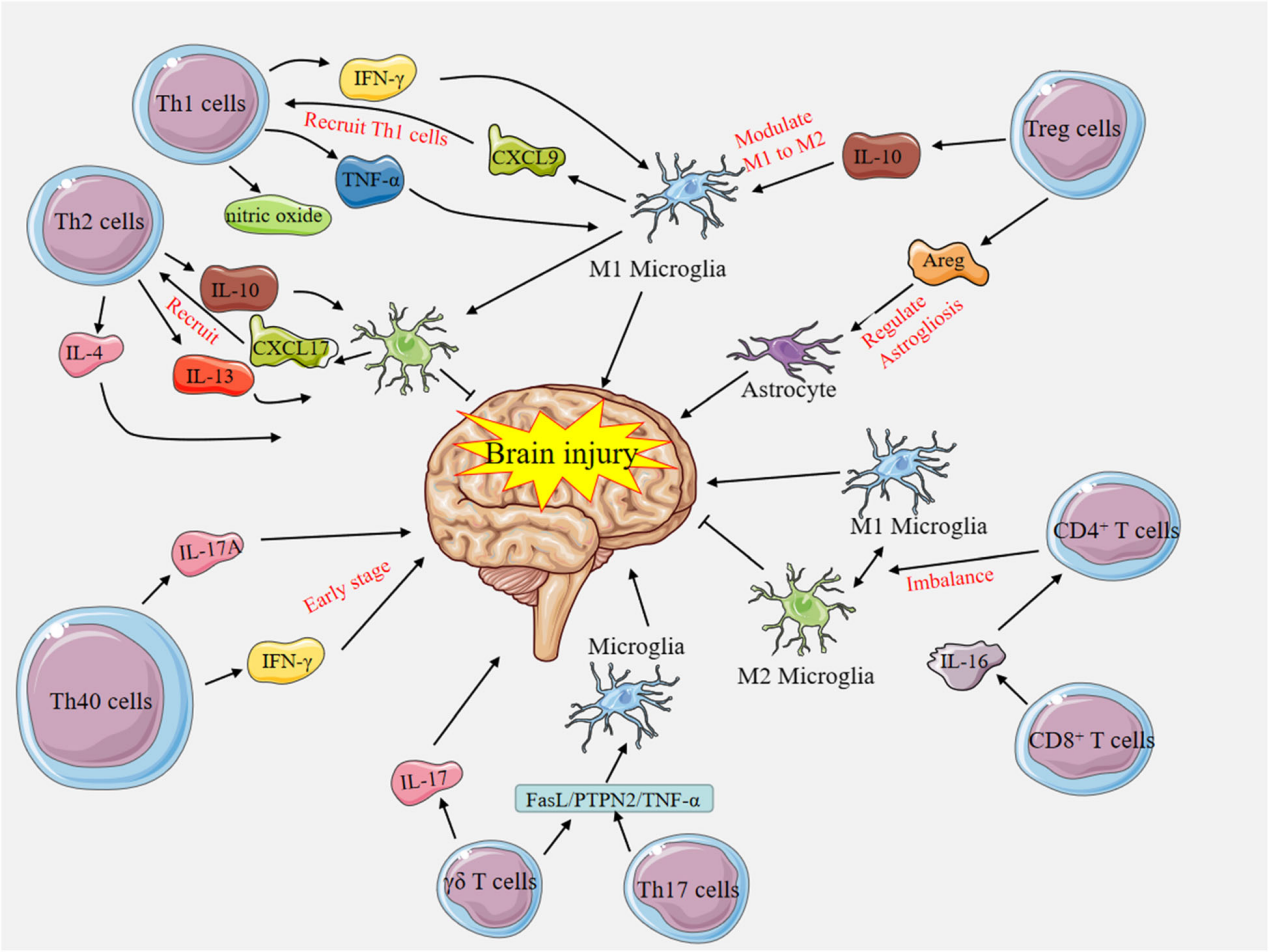
Different T cell subsets infiltrate the injured brain. Th1 cells secrete proinflammatory cytokines, such as IFN-γ and TNF-α, to promote M1 polarization. M1 microglia also induce and recruit Th1 cells by secreting IL-12 and TNF-α and expressing chemokines, such as CXCL9 and CXCL10. Moreover, Th2 cells secrete anti-inflammatory cytokines, such as IL-4, IL-10 and IL-13, to promote M2 polarization, and M2 cells can induce and recruit Th2 cells by secreting IL-4, CCL17, CCL22 and CCL24. Th40 cells, which are proinflammatory, secrete both IFN-γ and IL-17A and infiltrate the injured brain in the early stage after brain injury. γδ T cells secrete proinflammatory IL-17 to aggravate brain injury. Moreover, γδ T cells and Th17 cells activate proinflammatory microglia by modulating the FasL/PTPN2/TNF-α signalling pathway, which aggravates ischaemic brain injury. Treg cells interact with microglia and modulate microglial polarization from the M1 phenotype into the M2 phenotype via IL-10, and they also regulate astrogliosis by producing the cytokine amphiregulin (Areg). CD8^+^ T cells can recruit CD4^+^ mononuclear cells via the cytokine IL-16 after femoral artery ligation, and CD4^+^ T cells contribute to the imbalance in M1 and M2 polarization

#### Th40 cells related to brain injury

Deng et al. identified a new T cell subset, the Th40 cell subset, that is proinflammatory, secretes both IFN-γ and IL-17A and infiltrates the injured brain in the early stage (within 3 h) after CA/CPR or global cerebral ischaemia to contribute to neuronal injury [45]. Additionally, Th40 cell numbers increase again at 72 h, indicating a role in the sustained immune response [45]. Many studies have identified roles for these cells in autoimmune diseases, such as type 1 diabetes. Nonetheless, little is known about the roles of Th40 cells in the MCAO mouse model of ischaemia. More studies on Th40 cells in the injured brain after ischaemic stroke are needed.

#### Γδ T and Th17 cells related to brain injury

Shichita et al. proposed that γδ T cells, not Th17 cells, secrete proinflammatory IL-17 to aggravate I/R brain injury in the delayed phase (day 3) [109], although a previous study identified that IL-17-producing cell numbers peaked 3–5 days after injury in the ipsilateral cerebral hemispheres of patients following ischaemic stroke [110]. CD3^+^CD4^−^CD8^−^ T cells, also recognized as double-negative T cells (DNTs) and including γδ T cells, have been shown to coordinate immune and inflammatory homeostasis to exert functions in peripheral immune-related diseases [111, 112]. Subsequently, Meng et al. discovered that the number of DNTs was significantly elevated in a time-dependent manner, in both the injured brain and the peripheral blood, in both stroke patients and an MCAO mouse model. The experimental model showed that DNTs prominently infiltrated the injured brain from day 1 to day 3 after MCAO. The infiltrating DNTs activated proinflammatory microglia by orchestrating the FasL/PTPN2/TNF-α signalling pathway and then augmented cerebral immune and inflammatory responses to aggravate ischaemic brain injury [113]. Nonetheless, there are studies supporting the conclusion that Th17 cells play a detrimental role in the chronic phase of ischaemic stroke. These cells were found to exist in the injured brain at 1 week after traumatic brain injury (TBI) and drove the cytotoxicity of CD8^+^ T cells at the later stage, which potentiated the detrimental effects of CD8^+^ T cells seen in TBI [114, 115]. Likewise, increasing levels of IL-17 associated with worse neurological outcomes were found in the peripheral blood through 3 days after stroke onset in stroke patients [116, 117]. It remains to be clarified which types of immune cells are responsible for producing IL-17.

Studies have also found that γδ T cells link the innate immune response with the adaptive immune response. Because studies have shown that the acute adverse effects of T cells following acute ischaemic stroke are not associated with adaptive immune mechanisms, such as antigen recognition or costimulatory pathways [78], γδ T cells and natural killer T (NKT) cells may play detrimental roles in brain injury through the innate immune response.

#### Treg cells related to brain injury

Liesz et al. demonstrated that Treg cells were activated 5 days after MCAO and restrained to the peri-infarct zone, playing a protective role after brain injury [14]. Furthermore, Treg cells prevented secondary infarct growth by suppressing excessive production of proinflammatory cytokines and by orchestrating infiltration of lymphocytes and microglia, mainly via IL-10 signalling [14], in the ischaemic brain. Treg cells interact with microglia [100] and modulate microglial polarization from the M1 phenotype to the M2 phenotype via IL-10 [118]. Interestingly, a study showed that Treg cells gathered in the ipsilateral cerebral hemisphere during the chronic phase of ischaemic brain injury and regulated astrogliosis by producing the cytokine amphiregulin (Areg) [57]. Studies have also shown that Treg cells may adapt to different tissue environments by expressing distinct genes related to the tissue site and augment neurological recovery by suppressing neurotoxic astrogliosis by producing Areg in the context of the massive amplification and infiltration occurring in the chronic phase of stroke [119]. The 5-HT_7_ expression represents a specific way, one of hundreds, to amplify brain Treg cell functions [119]. Conversely, the function of Treg cells is controversial due to Treg cell depletion leading to a better outcome within 24 h and no progression until 1 week by ameliorating microvascular thrombus formation [120]. Additionally, Kleinschnitz et al. verified that Treg cells exerted a detrimental effect on mice with acute ischaemic stroke by inducing dysfunction in the cerebral microvasculature in the early phase [120]. In addition, the phenomenon of stroke-induced immunosuppression characterized by lymphopenia presents with a reduction in natural killer (NK) cell, B cell and T cell numbers in the peripheral blood and spleen and is thus vulnerable to bacterial infection, especially urinary tract infection and pneumonia in the subacute and chronic stages of ischaemic stroke [86]. This immunosuppression poses the question of whether adoptive transfer of Treg cells will create a more unbalanced immune state leading to an increase in the incidence of infection. Luckily, Li et al. revealed that Treg cells not only contribute to brain tissue protection and modulate CNS damage from a peripheral location but also regulate the homeostatic equilibrium of peripheral immune responses, such as simultaneously correcting immunosuppression and attenuating peripheral inflammation [121]. Previous literature has shown that Th cells possess plasticity, indicating that Th17 cells can transform into another T cell subset (such as Treg cells) within the injured brain during the process of neuroinflammation [69].

Nonetheless, neutrophils are still recognized as one of the first cells to infiltrate the injured brain, causing BBB disruption, cerebral oedema and brain injury, which indirectly prompt the infiltration of T cells [122]. In conclusion, more research is required to elucidate the biological roles of Treg cells to better understand how to modulate the immune system and facilitate healing after stroke.

#### Functions of CD4^+^ and CD8^+^ T cells related to stroke

Feng et al. found that chronic colitis exacerbated brain injury after stroke by inducing gut-derived CD4^+^ T cells to create an imbalance in M1 and M2 microglia/macrophages and increase the numbers of non-gut-derived CD4^+^ T cells infiltrating the brain [123]. CD4^+^ T cells have been identified to aggravate brain inflammation and induce neuronal death [100, 123]. Removal of the CD4^+^ T cell population attenuates apoptosis and enhances neurogenesis, while the elimination of CD25^+^ T cells, which include Treg cells, impairs functional recovery partly through the inhibition of neurogenesis after permanent experimental stroke [96]. Nonetheless, researchers have demonstrated that reducing astrogliosis and/or preserving neurogenesis play a vital role in protecting the injured brain during the repair stage after stroke [36, 119]. It is not clear which subsets of T cells are primarily responsible. Other researchers have identified that CD8^+^ T cells not only infiltrate the site of collateral vessel growth but also recruit CD4^+^ mononuclear cells to this site via the cytokine IL-16 after femoral artery ligation [124]. Interestingly, studies have found that stroke-induced immunodepression may be protective by reducing naive T cell and CD8^+^ CD45RA^+^ effector memory T cell (TEMRA) numbers to attenuate detrimental long-term antigen-specific immune responses in the CNS [125].

## Therapy related to T cells and stroke (Table 3)

### Cytokines, small molecules, neutralizing antibodies and cell epitopes as targets

We identified that IL-4 knockout (KO) mice have worse neurological outcomes than wild-type mice due to the increases in the Th1/Th2 ratio and Th1 polarization associated with greater injury [126]. Another study proposed that the source of IL-4 is neurons rather than T cells in tissue in the context of ischaemic brain injury, and the authors proposed that administering recombinant mouse IL-4 (rIL-4) subcutaneously could have a delayed role in protecting ischaemic brain tissue and improving outcomes, probably by polarizing the microglia into the healing M2 phenotype [148]. The specific mechanism remains to be clarified. Li and co-workers showed that astrocytic IL-15 could increase the severity of post-ischaemic brain injury by activating NK-, CD8^+^ T- and CD4^+^ T cell-mediated immunity [127]. Lee et al. identified that ablation of IL-15 using an anti-IL-15 neutralizing antibody decreased brain damage after ischaemic stroke by decreasing NK, CD8^+^ T and CD4^+^ T cell infiltration into the brain [128]. However, IL-15 was also reported to defend astrocytes against oxygen-glucose deprivation (OGD)-induced damage and death, and astrocytes could protect neurons from ischaemic injury and sustain BBB integrity [129], which are contradictory to IL-15 deficiency exerting a protective role in brain injury after ischaemic stroke. Clarkson et al. illustrated that treatments blocking T cell-derived IL-21 might improve neurological outcomes by reducing lymphocytic brain infiltration and attenuating neuronal autophagy [149]. Xiao et al. found that pretreatment with IL-33, a new member of the IL-1 cytokine family, improved neurological outcomes by suppressing the Th1 cell response and improving the Treg cell response in mice, indicating that IL-33 might play a long-term protective role by modulating peripheral immune responses after ischaemia [130, 150].

**Table 3:** Stroke therapy related to T cells

Target therapy		Experimental model	Function	Molecular mechanism	Reference
Cytokines, small molecules, neutralizing antibodies, cell epitopes					
IL-4/rIL-4 injected subcutaneously		Mouse model	Increasing Th2 cells and promoting polarization of microglia to the healing M2 phenotype	Exerting the function of IL-4	Zhao et al. [124]
IL-15/IL-15 neutralizing antibody injected subcutaneously		Mouse model	Decreasing NK, CD8^+^ T and CD4^+^ T cells infiltrating the brain	Exerting the function of IL-15	Lee et al. [126]
IL-21/IL-21 receptor Fc protein injected intraperitoneally		Mouse model	Blocking T cell-derived IL-21 to reduce CD4^+^ and CD8^+^ cells infiltrating the brain and attenuate neuronal autography	Exerting the function of IL-21	Clarkson et al. [127]
IL-33/IL-33 injected intraperitoneally		Male mouse model	Suppressing Th1 cell response as well as improving Treg cell response	Downregulating the expression of the transcription factor T-bet and upregulating the expression of GATA-3 and Foxp3	Xiao et al. [128]
PD-1/humanized anti-PD-L1 antibody		Mouse model/clinical trial	Increasing the appearance of CD8^+^ regulatory T cells in the lesioned brain and decreasing CNS infiltrating immune cells	Unclear	Bodhankar et al. [129], Zhang et al.
DHA/DHA injected intraperitoneally		Mouse model	Attenuating the infiltration of T cells into injured brain tissue and promoting polarization of microglia to the healing M2 phenotype	Reducing the production of CCL3, CCL17, CXCL10 and CXCL12 to decrease the quantity of T cells	Cai et al. [130]
GSF/GSF injected intraperitoneally		Rat model	Attenuating the recruitment of T cell in post-stroke injured brain	Reducing blood-brain barrier disruption	Dietel et al. [131]
CXCL14/2-methoxyestradiol injected intraperitoneally		Rat model	Inducing Treg differentiation	Promoting accumulation of iDC to secrete IL-2 to induce Treg differentiation	Lee et al. [132]
ACC1/(caloric restriction)		Mouse model	Balancing peripheral regulatory T cells/T helper 17 (Th17) cells	Inhibiting the ACC1 enzyme	Wang et al. [133]
CD28/CD28SA injected intraperitoneally		Mouse model	Expanding and amplifying Treg cells that produce IL-10	Boosting the production of IL-10	Na et al. [134]
TLR/The antibodies of TLR2, TLR4 and TLR8		Vitro study	Reducing the activation of T cells	Unclear	Tang et al. [135]
RTLs/RTL551, RTL100 ] injected subcutaneously		Male DR2-Tg mice	Inhibiting the activation or infiltration of CD3^+^ T cells and other proinflammatory cells	Modulating T cell functional properties and blocking immune cells infiltrating the brain	Zhu et al. [136]
Glycyrrhizin (Gly)/injected intraperitoneally		Mouse/rat model	Inhibiting the activation of CD8^+^ T and CD4^+^ T cells	Inhibiting HMGB1 release, which promoted T cell proliferation	Xiong et al. [137]
Exogenous vitamin D3/injected intraperitoneal injection		Mouse model	Reducing Th17/γδ T cell response and increasing Treg cell response	Reducing the expression of proinflammatory mediators IL-6, IL-1β, IL-23a, TGF-β and NADPH oxidase-2 and expression of the transcription factor, ROR-γ	Evans et al. [138]
Cells					
Intravenous cellular/injected intravenously (MAPCs)		Animal model/clinical trial	Reducing proinflammatory cells including CD3^+^ T, CD4^+^ T and CD8^+^ T cells and promoting Tregs	Relating to multiple mechanisms of action	Mays et al. [139]
Treg					
Treg/antibiotic-induced intestinal flora alteration		Mouse model	Increasing regulatory T cells and reducing IL-17^+^ γδ T cells	Altering dendritic cell activity to induce Treg cell differentiation more effectively	Benakis et al. [140]
Treg/adoptively transferred Treg		Mouse model	Increasing the number and/or function of Treg	Unclear	Xia et al. [141]
Brain antigen/intranasal instillation	MBP	Male rat model	Suppressing Th1 response and increasing the probability of Tr1, Th3 or other Tregs responses	Inducing mucosal tolerance	Gee et al. [142]
	E-selection	SHR-SP rat model			Chen et al. [143]
	MOG	Female rat model			Frenkel et al. [144]
Drugs					
Levodopa/benserazide/injected intraperitoneally		Rat model	Reducing CD8^+^ cells infiltrating the injured brain	Reducing the expression of ICAM-1 on endothelial cells in the brain to inhibit adhesion of cytotoxic T cells infiltrating the brain parenchyma	Kuric et al. [145]
Natalizumab/injected intravenously		Clinical trial	Blocking T cell infiltration into the brain	Blockade of the α4-β1 integrin on leukocytes	Veltkamp et al. [146], Fu et al. [147]
Fingolimod/orally		Clinical trial	Reducing peripheral lymphocytes	An oral S1P receptor modulator that sequesters lymphocytes to lymph nodes	Veltkamp et al. [131], Fu et al. [147]

Bodhankar et al. identified that PD-1 and CTLA-4 had inhibitory effects on the activation of T cells in a rodent stroke model [151] and that blockade of the PD-L1 checkpoint significantly limited the CNS inflammatory response and improved neurological outcomes by partially reversing splenic atrophy and increasing the accumulation of CD8^+^ Treg cells in the lesioned brain hemisphere [131]. This suggests the application potential of a novel therapy using accessible humanized anti-PD-L1 antibodies to treat human stroke subjects and confirms that PD-1 is inversely correlated with the absolute amount of CD4^+^ T central memory (TCM) cells in ischaemic patients [135]. However, these conclusions remain to be validated in clinical trials.

Systemic administration of docosahexaenoic acid (DHA), a major form of omega-3 polyunsaturated fatty acids (n-3 PUFAs) in the CNS, may reduce post-stroke brain injury by attenuating T cell infiltration, thereby decreasing the immune response in injured brain tissue and promoting the polarization of macrophages into the healing M2 phenotype [133].

Granulocyte colony-stimulating factor (G-CSF) was reported to have immunomodulatory effects and suppress the migration and maturation of dendritic cells (DCs) to exert neuroprotective effects [136]. The administration of a single dose of G-CSF can attenuate the recruitment of T cells to the injured brain following stroke, which has positive effects [140].

Toll-like receptors (TLRs), especially TLR2 and TLR4, have been broadly reported to play detrimental roles following ischaemic stroke, and TLR2-deficient and TLR4-deficient mice have been shown to have neurological function protection after ischaemic stroke mediated by attenuation of the activation of T cells. TLR8 may produce the same effect [141]. Inhibition of acetyl coenzyme A carboxylase 1 (ACC1), which has been achieved by either conditional knockout or pre-treatment with caloric restriction, is a novel approach to balance Treg cells and Th17 cells and has a protective effect on brain injury after ischaemic stroke [142].

Recombinant T cell receptor ligands (RTLs) have been studied to find a novel target to improve neurological outcomes after ischaemic stroke [152]. Zhu et al. demonstrated that in addition to RTL551, RTL1000 could improve long-term neurological outcomes following ischaemic stroke by inhibiting the activation or infiltration of CD3^+^ T cells and other proinflammatory cells [152].

### Treg cells as a therapeutic target

Treg cells may improve stroke outcomes by suppressing IL-17^+^ γδ T cell proliferation by altering the intestinal flora rather than being present in the brain [153]. Directly augmenting Treg cell numbers through adoptive transfer has been shown to be an efficacious way to protect the injured brain and promote long-term recovery after stroke [144]. However, there are many issues with this approach, such as the requirement for ex vivo-expanded Treg cells and the aggravation of stroke-induced immunosuppression [144]. Numerous studies have demonstrated that inducing a Treg cell response to a brain antigen, such as myelin basic protein (MBP) [154], E-selectin [143] or myelin oligodendrocyte glycoprotein (MOG) [132, 155], through intranarial instillation can improve neurological outcomes via the “bystander suppression” approach. “Bystander suppression” is defined as an immune response in which Treg cells are stimulated in an antigen-specific manner but secrete cytokines modulating immune responses in an antigen-nonspecific manner, implying that a therapeutic immunomodulatory response can be induced regardless of whether the pathogenic antigen is known [154]. The immunomodulatory response is thought to occur through enhancement of a Th3 (TGF-β)-type response or other Treg response and suppression of the Th1 response or other immune responses [134, 154, 155] and may promote adult neurogenesis after ischaemia [137]. However, tolerization to antigens such as MBP prior to ischaemia may cause detrimental autoimmunity via the development of a Th1 response to the antigen by 3 months after ischaemia [63]. Other studies have illustrated that passive CXCL14 supplementation improves neurological deficits after ischaemic stroke by promoting immature dendritic cell (iDC) secretion of IL-2, which induces Treg cell differentiation and other positive pathways [138]. A super-agonistic anti-CD28 monoclonal antibody (CD28SA) can expand and amplify Treg cells that produce IL-10 to attenuate brain damage after ischaemic stroke [145]. However, both of the clinical trials (NCT00012454 and NCT00069069) evaluating E-selectin nasal instillation have failed.

### Using drugs for therapy

Some immunomodulatory drugs have shown promise as novel therapies that decrease morbidity and mortality following ischaemic stroke. Glycyrrhizin (Gly) is thought to protect against brain damage induced by ischaemic stroke by inhibiting the activation of CD8^+^ and CD4^+^ T cells mediated by IFN and partly regulated by HMGB1 activity [147]. Administration of exogenous vitamin D3 prior to stroke may improve neurological deficits and produce an acute anti-inflammatory response by reducing the Th17/γδ T cell response and increasing the Treg cell response [146]. Levodopa/benserazide treatment after stroke onset was shown to play a protective role by reducing CD8^+^ cell infiltration into the injured brain [139]. Blocking α4 integrin on leukocytes with natalizumab can provide delayed protection in a mouse model [81], but there is not enough evidence that it is effective in clinical trials [156]. Therefore, the protective role of natalizumab in determining functional outcomes in ischaemic stroke requires further clinical research. Fingolimod, an oral S1P receptor modulator used to reduce peripheral lymphocyte numbers [157], was shown to enhance short-term and long-term neurological recovery in clinical trials with or without alteplase [158–160]. These two drugs are very promising for the future treatment of ischaemic stroke [161, 162].

### Using intravenous cells for therapy

Intravenous cellular therapies have intrigued many researchers and clinicians over the past decades because of their potential advantage of affecting immune responses through multiple mechanisms and actions [163]. Both animal stroke models and a multi-arm phase 2 clinical trial have shown that intravenous injection of multipotent adult progenitor cells (MAPCs) enhances long-term neurological recovery by modulating immune responses. The underlying mechanism may involve reducing the levels of proinflammatory cells such as CD3^+^, CD4^+^ and CD8^+^ T cells while promoting Treg cell accumulations [163].

Despite discrepancies and heterogeneity amongst studies, new therapeutic targets that can balance the immune response of T cells to protect against the acute and chronic phases after ischaemic stroke are being discovered.

## Conclusion

Despite the various types and functions of T cells, most studies have focused on common T cell subsets, including Th1, Th2, Th17, γδ T and Treg cells. These cells intricately communicate with each other and with injured brain tissue via proinflammatory cytokines and anti-inflammatory cytokines and perform immunomodulatory roles. While the inconsistent description of the roles of T cells may be partly due to the differences in stroke models and measurement methods, as well as discrepant post-stroke outcomes, the influx of different subsets of T cells at different stages after ischaemic stroke requires more study. Nevertheless, the evidence reviewed here demonstrates that the interactions of T cells with the CNS and the connections of these cells with other immune cells are complicated and need further elaboration [164]. In conclusion, although a number of studies have elucidated that T cell numbers peak in the infarct zone and peri-infarct zone within 30 days after ischaemic stroke, T cells play an indispensable long-term role after ischaemic stroke through mechanisms such as tissue remodelling and revascularization [165] and therefore are a new target for clinical stroke treatment.

Future research must not only examine how the immune response mediated by T cells is initiated and maintained but also differentiate the various roles of T cell subsets in the onset and process of post-stroke tissue injury and repair. These studies could inform approaches for designing immunoregulatory therapies that regulate T cells in the acute stage following stroke to improve the functional outcome and long-term sequelae of patients suffering from ischaemic stroke. Although current studies have identified a large number of targets, such as cytokines, small molecules, neutralizing antibodies, cell epitopes and injectable cellular products, to regulate the immune response and inflammation related to T cells in the acute or chronic phase following stroke, the most important issue is whether a further understanding of T cell inflammation will provide more comprehensive therapeutic targets and lead to successful clinical translation of immune modulators for stroke. The results of previous studies that manipulated T cell responses have not been completely clarified. The protective effect of suppressing T cells in the acute phase may be based on attenuating neuroinflammation, and long-term protection may refer to the role of Treg cells in tissue repair. However, no comprehensive clinical trial has demonstrated the clinical efficacy or safety of these treatments. Nonetheless, great effort is being put into exploring the underlying mechanisms of these therapies. We still have many challenges to overcome in the pursuit of understanding the pathogeneses and therapies of ischaemic stroke.

We need to perform more studies to understand the roles and mechanisms of T cells in the onset and evolution of ischaemic stroke and to further explore the modulation of both local and peripheral T cell responses, with the goal of attenuating acute neuroinflammation and improving long-term neurological function following ischaemic stroke.

## Data Availability

Not applicable

## Abbreviations

DAMPs: Damage-associated molecular patterns
CD: Cluster of differentiation
TCR: T cell receptor
Th cells: T helper cells
STAT: Signal-transducing activator of transcription
IFN: Interferon
IL: Interleukin
Treg cells: Regulatory T cells
NKT cells: Natural killer T cells
IRF: Interferon-regulatory factor
TGF-β: Transforming growth factor-β
GM-CSF: Granulocyte macrophage colony-stimulating factor
EAE: Experimental autoimmune encephalomyelitis
TNF: Tumour necrosis factor
AHR: Aryl hydrocarbon receptor
CCR: C-C chemokine receptor
APCs: Antigen-presenting cells
RA: Rheumatoid arthritis
NBNT: Non-B/non-T
NF-κB: Nuclear factor kappa-light-chain-enhancer of activated B cells
Tnfrsf: TNF receptor super family
TRAFs: TNF receptor-associated factors
CA/CPR: Cardiac arrest and cardiopulmonary resuscitation
Tfh: Follicular helper T cells
CXCR: C-X-C chemokine receptor
CXCL: Chemokine (C-X-C motif) ligand
Bcl6: B cell lymphoma 6 protein
c-Maf: Cellular muscular aponeurotic fibrosarcoma
Batf: B cell-activating transcription factor
Blimp1: B lymphocyte-induced maturation protein-1
S1PR2: Sphingosine-1-phosphate receptor-2
GCs: Germinal centres
LPS: Lipopolysaccharide
ICOSL: Inducible co-stimulator ligand
Rag: Recombination activation gene
SCID: Severe combined immunodeficiency
tMCAO: Transient middle cerebral artery occlusion
pMCAO: Permanent middle cerebral artery occlusion
CNS: Central nervous system
ROS: Reactive oxygen species
BBB: Blood-brain barrier
CCL: Chemokine (C-C motif) ligand
I/R: Ischaemia-reperfusion
DNTs: Double-negative T cells
Fas ligand: FasL
Areg: Amphiregulin
TEMRA: CD45RA+ effector memory T cell
IL-4 KO: IL-4 knockout
rIL-4: Recombinant mouse IL-4
NK: Natural killer
OGD: Oxygen-glucose deprivation
PD-1: Programmed cell death-1
CTLA: Cytotoxic T lymphocyte antigen
PD-L1: PD-ligand1
TCM: T central memory
DHA: Docosahexaenoic acid
n-3 PUFAs: Omega-3 polyunsaturated fatty acids
G-CSF: Granulocyte-colony stimulating factor
DCs: Dendritic cells
TLRs: Toll-like receptors
ACC1: Acetyl coenzyme A carboxylase 1
RTLs: Recombinant T cell receptor ligands
MBP: Myelin basic protein
MOG: Myelin oligodendrocyte glycoprotein
iDC: Immature dendritic cell
CD28SA: Super-agonistic anti-CD28 monoclonal antibody
Gly: Glycyrrhizin
HMGB1: High-mobility group box 1
MAPCs: Multipotent adult progenitor cells
